# Monitored Implementation of COVID-19 Rapid Antigen Screening at Taxi Ranks in Johannesburg, South Africa

**DOI:** 10.3390/diagnostics12020402

**Published:** 2022-02-03

**Authors:** Mohammed Majam, Vanessa Msolomba, François Venter, Lesley Erica Scott, Trish Kahamba, Wendy Susan Stevens, Michael Rademeyer, Tanya van Tonder, Sanjida Karim, Rigveda Kadam, Paula Akugizibwe

**Affiliations:** 1 Ezintsha, Faculty of Health Sciences, University of the Witwatersrand, Johannesburg 2193, South Africa; vmsolomba@ezintsha.org (V.M.); fventer@ezintsha.org (F.V.); 2 Department of Molecular Medicine and Haematology, University of the Witwatersrand, Johannesburg 2193, South Africa; lesley.scott@wits.ac.za (L.E.S.); trish.kahamba@ilead.org.za (T.K.); wendy.stevens@wits.ac.za (W.S.S.); 3 The National Priority Program of the National Health Laboratory Service, Johannesburg 2193, South Africa; 4 A2D24, Albury Office Park, 5 Albury Rd., Dunkeld West, Randburg 2196, South Africa; michael_rademeyer@a2d24.com; 5 Opinion Solutions, Melrose Arch, 44 Melrose Blvd, Birnam, Johannesburg 2196, South Africa; tanya@opinionsolutions.co.za; 6 FIND, Campus Biotech, Chemin des Mines 9, 1202 Geneva, Switzerland; Sanjida.karim@finddx.org (S.K.); Rigveda.Kadam@finddx.org (R.K.); Paula.Akugizibwe@finddx.org (P.A.)

**Keywords:** COVID-19, SARS-CoV-2, testing, antigen, rapid, taxi rank, digital, South Africa, differentiated care, community-based testing

## Abstract

Digital tools can support community-based decentralized testing initiatives to broaden access to COVID-19 diagnosis, especially in high-transmission settings. This operational study investigated the use of antigen-detecting rapid diagnostic tests (Ag-RDTs) for COVID-19 combined with an end-to-end digital health solution, in three taxi ranks in Johannesburg, South Africa. Members of the public were eligible if they were aged ≥18 years, could read, and had a cellphone. Over 15,000 participants, enrolled between June and September 2021, were screened for COVID-19 risk factors. A digital risk questionnaire identified 2061 (13%) participants as moderate risk and 2987 (19%) as high risk, based on symptoms and/or recent exposure to a known case. Of this group referred for testing, 3997 (79%) received Ag-RDTs, with positivity rates of 5.1% in the “high-risk” group and 0.8% in the “moderate-risk” group. A subset of 569 randomly selected participants received additional PCR testing. Sensitivity of the Ag-RDT in this setting was 40% (95% CI: 30.3%, 50.3%); most false negatives had high cycle threshold values (>25), hence low viral loads. Over 80% of participants who tested positive completed a 2-week phone-based follow-up questionnaire. Overall, the digital tool combined with Ag-RDTs enhanced community-based decentralized COVID-19 testing service delivery, reporting and follow-up.

## 1. Introduction

The COVID-19 pandemic, caused by the SARS-CoV-2 virus, has proven difficult to control, particularly during periodic peaks and waves of infection. South Africa has reported around 3.46 million COVID-19 cases and 91,000 directly associated deaths as of December 2021 [1]. South Africa’s second wave of COVID-19, which peaked in January 2021, saw the emergence of the Beta variant as the predominant strain of the virus [2]. A third wave of COVID-19 started in the country in June 2021 dominated largely by the Delta variant, followed by the emergence of the Omicron variant in November 2021 [2,3]. The pandemic has had a substantial impact on the lives and livelihoods of South Africans, and has impacted access to healthcare services, through restrictions on public movement and diversion of healthcare resources to the pandemic response [4]. Difficulties in accessing timely COVID-19 tests, vaccines and treatments have also complicated control of COVID-19 in the country.

In South Africa, community spread of COVID-19 during waves of the pandemic has been high, and mostly brought on by confounding factors such as socioeconomics, crowded living quarters, a heavy reliance on public transport, and social gatherings, such as church events and funerals. Multi-person minibus taxis are the most common mode of public transport in densely populated areas such as Johannesburg, the economic hub of the country. With close to 15 million commuter trips daily in South Africa [5], taxis are potentially important vectors in disease spread. Whilst there has not been a study into COVID-19 transmission in taxis, previous modelling work has indicated that the environmental risk associated with lack of adequate ventilation in taxis contributes to sustained transmission of other infectious diseases like tuberculosis [6]. Taxi ranks are also often high foot traffic areas, closely associated with various forms of trade in and around the vicinity of the rank, making them prime sites for decentralized testing interventions.

In such settings with a high potential for widespread community transmission, testing for COVID-19 is a critical tool to control the spread of the virus. Widespread testing, when deployed effectively, may enable the identification of infected individuals and the subsequent implementation of test-trace-isolate strategies, which can help break chains of transmission, through informing individuals of their status and methods to limit their transmission risk. Numerous diagnostic products to detect active SARS-CoV-2 infection are now on the market, broadly split into the rapid antigen-detecting tests (Ag-RDTs) that can be used at or near the point of care, and laboratory-based nucleic acid amplification testing (NAAT) approaches, such as reverse transcription polymerase chain reaction (RT-PCR) [7].

While RT-PCR is considered the gold standard for COVID-19 testing [7], such services have often been difficult to access, especially outside of metropolitan areas in low- and middle-income countries (LMICs) or backlogged for several days to weeks, particularly during new waves of the pandemic. In addition, PCR tests require sophisticated laboratory infrastructure, are expensive [8], and can remain positive long after the period of infectiousness [9]. Reliance on the centralized laboratory infrastructure required for PCR can also deter patients from getting tested, particularly if they have to travel to health facilities to access diagnostic services. Evidence from other disease response, such as HIV, has highlighted the importance of decentralized diagnosis through the use of point-of-care technologies, in order to improve outcomes [10].

Lateral flow format Ag-RDTs, which are easy to perform and typically provide results within 15–30 min have the potential to fill at least a portion of the testing gap. These simple-to-use tests offer the possibility of rapid case detection, especially of the most infectious patients, at or near the point of care [11,12]. Ag-RDTs meeting minimum performance standards have been recommended by the World Health Organization as testing strategies in certain scenarios where they are likely to have the most impact on early detection of cases for care and contact tracing and where test results are most likely to be correct [8]. Further, adding Ag-RDT testing to supplement the overall testing strategy has the potential to be highly cost effective, driven by the lower price point for the test and the ability to test at the point of care, which can reduce potential transport costs [13]. 

National policies are being adapted in many countries to allow and encourage targeted use of these Ag-RDTs [14]. Around the world, Ag-RDTs are being used across a range of use cases including in schools, workplaces and at public events [15,16,17,18]. Some high-income countries, like the United Kingdom and Canada, have also introduced initiatives to make Ag-RDTs widely available [19,20]. However, access to Ag-RDTs is more limited in many LMIC settings [14], where the need for them may be even greater given the previously mentioned challenges with PCR diagnosis.

In South Africa, PCR remains the mainstay of COVID-19 diagnosis, but uptake of Ag-RDTs has gradually increased since their introduction in October 2020. As of 4 December 2021, they accounted for 17% of all tests conducted in the country since the start of the pandemic [21]. As part of efforts to control ongoing COVID-19 waves, targeted use of Ag-RDTs for decentralized testing in high-transmission settings may be a resource-efficient approach in South Africa and other countries.

However, testing in decentralized settings requires establishing systems for data management and patient follow-up outside of healthcare facilities, which can be a challenge. Adequate monitoring of services to ensure standardized approaches in line with national guidance, and provide mentoring or troubleshooting where needed, can also be more challenging when services are being delivered in community settings.

Digital technology-based approaches that support testing—including related data management and patient follow-up functions—can help address these operational challenges, and have shown considerable promise in increasing access to and the efficiency of testing for COVID-19 and other diseases [22,23,24,25,26]. The value of digital health solutions in decentralized testing settings such as transport hubs and taxi ranks has also been demonstrated through programmes such as HIV Self-Test Africa (STAR) in South Africa [27]. Digital tools can facilitate a standardized approach to decentralized testing, and strengthen reporting, which can in turn help reduce errors, improve the acquisition of vital patient data, facilitate linkage to treatment, and reduce human resources and costs. These features are particularly advantageous when it comes to large-volume testing in high-throughput scenarios, such as community-based screening of COVID-19.

Evidence from the field is needed to help understand how Ag-RDTs can be best used as part of decentralized testing initiatives in lower-resource settings, and whether digital solutions can enable effective data and patient management. With WHO estimating that six out of seven COVID-19 cases in the African region go undetected, such initiatives could prove key to improved management of the pandemic in this region [28].

Given the need to generate evidence that can inform initiatives to improve COVID-19 diagnosis in LMICs, and the significance of taxi ranks as potential high-transmission sites, this study set out to conduct monitored implementation of Ag-RDTs for COVID-19 screening in conjunction with an end-to-end digital health solution in three taxi ranks in Johannesburg, South Africa. In this paper, we share how this testing intervention was implemented and study findings, in terms of the outcomes of individuals who accessed testing through this intervention, the utility of the tool in supporting screening, data management and patient follow-up, performance of the Ag-RDT in the setting, and operational learnings to guide future decentralized testing interventions.

## 2. Materials and Methods

### 2.1. Study Design and Population

This was a prospective evaluation of the performance and operational characteristics of SARS-CoV-2 Ag-RDTs in combination with a digital health solution in three transport hubs in Johannesburg. The study protocol is registered on the South African National Clinical Trials Registry (Trial Number: DOH-27-072021-7413). 

Eligible participants included commuters, drivers, and vendors ≥18 years of age frequenting the three high foot traffic taxi ranks in Johannesburg (Baragwanath, Randburg and Germiston taxi ranks). Participants had to provide consent to participate in the study and had to be able to read. Participants also needed a cellphone capable of receiving Unstructured Supplementary Service Data (USSD), SMS (text message), or WhatsApp messaging. Key exclusion criteria included any contraindications to nasopharyngeal sample collection (e.g., recent nasal trauma), vulnerable populations deemed inappropriate for the study, personnel directly involved in the study, and anyone with a confirmed COVID-19 diagnosis within three months of re-testing. 

### 2.2. Study Objectives

The primary objective of the study was to use a digital solution in conjunction with Ag-RDTs to support testing at Baragwanath, Randburg and Germiston taxi ranks. Specific primary objectives were to screen participants using the digital algorithm built into the tool to determine COVID-19 risk, conduct Ag-RDT testing for all suspect cases (those assigned medium and high risk by the algorithm), and provide quick results to participants. 

The study also investigated a number of secondary objectives, which included:

1. Providing digital follow-up for participants in terms of the proportion of low-risk participants that completed at least one follow up survey and the proportion of positive participants that completed the 10-day post-diagnosis follow up questionnaire;
2. Determining the SARS-CoV-2 positivity rate among suspect cases tested using Ag-RDTs during the project by occupation;
3. Determining the positivity rate among randomly selected individuals tested using PCR during the project by occupation;
4. Measuring the field performance of Ag-RDT testing in this setting, in terms of the proportion of individuals testing positive with Ag-RDTs who are true positives according to PCR; and
5. Assessing participants’ level of satisfaction with the digital solution, as determined by a post-intervention questionnaire.

### 2.3. Digital Health Tool

The digital health tool was developed as a smartphone and tablet application that provides an end-to-end digital pathway for Ag-RDT testing in the taxi ranks, encompassing screening and risk identification, sample collection and capturing of demographic information, as well as subsequent reporting of results. The tool was developed by Ezintsha in conjunction with software developers A2D24, Johannesburg, South Africa. It also includes a back-end dashboard that produces real-time analytics on study indicators and any other variables or relationships of interest, to support interpretation of findings as well as operational management.

### 2.4. Study Process

**Figure 1:**
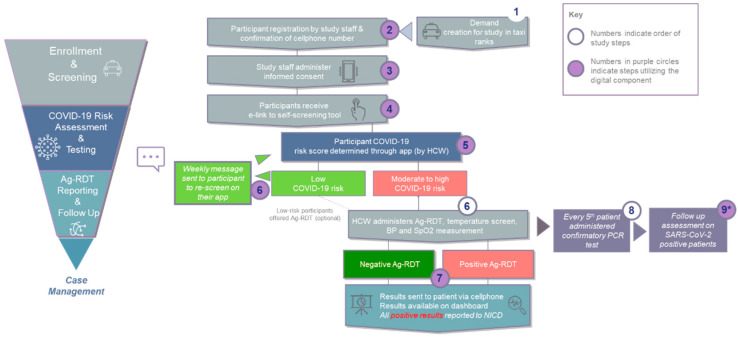
Overall study process. * Participants whose PCR results were discordant from their Ag-RDT results were notified and updated results were sent to NICD. Ag-RDT, antigen-detecting rapid diagnostic test; BP, blood pressure; HCW, healthcare worker; NICD, National Institute for Communicable Diseases; PCR, polymerase chain reaction.

The overall process for the study is shown in Figure 1 and described below.

#### 2.4.1. Study Team Composition

Each of the three sites was staffed with four to six recruiters, two healthcare workers who captured demographics and screened participants, and two nurses (one professional and one registered) to conduct testing. 

#### 2.4.2. Demand Creation 

Prior to accessing the taxi ranks, the study team met with taxi rank operators to explain the rationale for the study, get their buy-in and negotiate spatial planning and timing. Two weeks prior to initiation of testing, fieldworkers were deployed to create awareness through word of mouth, and distribution of pamphlets, posters and signage throughout the ranks. During the three-month testing period, commuters, drivers and vendors in the ranks were approached by study outreach personnel and invited to participate. Depending on the individual’s language preference, details about the project were explained verbally in common local languages/dialects (either English, Sepedi, Sesotho, Setswana, Ndebele, Isixhosa or Zulu). Because of the diversity of the province, engagement options in multiple languages were required.

#### 2.4.3. Enrollment and Screening 

Testing tents and booths (gazebo structures) were set up at each of the participating taxi ranks, with queue management to reduce congestion at the testing site. Consenting participants were screened on-site by trained healthcare workers to confirm eligibility for the study and then registered using the app. During the registration process, the study team captured patient demographics in the app and attempted to validate the participant’s cellphone number through SMS. Each participant was issued a unique identifier for use throughout the testing and reporting process. 

#### 2.4.4. Risk Assessment and Testing

Trained recruiters and healthcare workers assessed each participant’s risk of COVID-19 using the questionnaire on the digital tool (available in Supplementary Figure S1). Based on responses, participants were assigned to risk categories using South Africa’s National Department of Health’s definitions [29]. Risk factors were either symptoms and/or recent exposure to a known case and did not include pre-existing conditions that may predispose an individual to complications from COVID-19 (e.g., asthma). High risk was defined as two or more COVID-19 related risk factors on the questionnaire, moderate risk was one risk factor, and low risk was no risk factors. Participants determined to be moderate or high risk were invited to enrol as testing participants but all individuals who registered for the study were offered testing with an Ag-RDT if they wished, regardless of risk profile.

At the time of the study, only two Ag-RDTs were approved for use in South Africa, of which only one had readily available supplies: the Panbio™ COVID-19 Ag Rapid Test Device (Abbott, San Diego, Carlsbad, CA, USA), which detects the nucleocapsid (N) protein of SARS-CoV-2 [30]. The Panbio Ag-RDT can detect all major SARS-CoV-2 Variants of Concern [31,32]. Ag-RDTs were performed by trained healthcare workers according to the manufacturer’s instructions and according to the national policy on all individuals referred for testing. All participants found to be SARS-CoV-2-positive through the Ag-RDT were reported to the National Institute for Communicable Diseases. Participants were allowed to get tested more than once, if they experienced new or worsening symptoms during the study, following a negative test. 

Specimens for confirmatory PCR testing were taken from every fifth participant presenting for testing regardless of the Ag-RDT result, for quality control purposes and to ascertain concordance between the two testing platforms. Samples were sent to the Clinical Laboratory Services Lab (Department of Molecular Medicine and Haematology, University of the Witwatersrand, Johannesburg, South Africa) for PCR, performed as per existing standard operating procedures (QuantStudio 5 Real-Time PCR System, Firmware version 1.3.3) using the TaqPath SARS-CoV-2 (Thermo Fisher Scientific, Waltham, MA, USA) assay. 

#### 2.4.5. Reporting and Follow-Up

Results from the Ag-RDTs were captured in the digital tool and then sent directly to the participant’s phones via WhatsApp and SMS. PCR results positive for SARS-CoV-2 or discordant with the Ag-RDT result were sent via SMS to the participant’s phone. Participants with positive results were counselled and followed up for 14 days to understand isolation, contacts, disease severity (graded on a Likert scale), and resolution of infection or hospitalization. Participants with negative results were followed up via message to prompt them to complete the screening questionnaire weekly on their own devices.

Three follow-up approaches were used, depending on the participant’s risk and sero-status:

1. Participants assigned as low risk were registered to receive weekly messages reminding them to repeat the symptom screening and return for a test if their risk status changed over the study period.
2. Participants who were assigned as moderate/high risk but did not present for testing were followed up by a call centre agent. During the call, their reasons for not completing a test were also documented. After three unsuccessful attempts to contact the participant, the individual was considered lost to follow up.
3. Participants testing positive were sent a daily symptom screening for 10 days, and after 14 days were sent a message to respond to regarding resolution of symptoms. Participants were actively called through a call centre agent employed by the project to be assisted with further linkage to care services if their COVID-19 symptoms did not resolve after 14 days or if the participant did not respond to the Day 14 message.

### 2.5. Assessing Commuter Perspectives and Practices on SARS-CoV-2 Testing

A rapid phone-based survey was also conducted among random commuters in the taxi ranks where testing was conducted, to understand general perceptions and practices around testing, before and after the intervention. This survey was not specific to study participants and respondents were not formally enrolled, but approached by field workers to submit anonymous responses, so as to gain broader context when interpreting study findings. Standardized questionnaires were used and responses were captured on a mobile device.

### 2.6. Sample Size and Analysis 

The study aimed to screen 13,500 participants, across the three taxi ranks over a period of three months. Based on previous work in taxi ranks, the majority of participants were expected to be from the general population transiting through the taxi rank, with less than 7% from drivers and vendors. Sample size was not powered to evaluate any specific objective, but rather based on feasibility considerations given the time and budget available for testing. Based on estimated prevalence of COVID-19 at the time of recruitment, it was assumed that at least 5000 moderate- and high-risk individuals would be identified through the screening process. 

Demographic characteristics (age, gender, location) of each study group were tabulated, along with the median age (plus range and standard deviation) of the enrolled participants, as a whole and stratified by gender and group. Participants were also stratified by taxi rank and type of participant (driver/vendor/general population).

### 2.7. Ethical Considerations 

All participants had to provide informed consent to participate in the study, by signing an electronic or paper-based consent form. Participants had the right to withdraw from the study at any time. The study was conducted according to ICH Guidelines and South Africa legal requirements regarding research. The study was approved by the Human Research Ethics Committee responsible for oversight of research conducted at the study site. The Investigator maintained paper or electronic source documentation for all study participants, and all identifying information was kept confidential and de-identified.

## 3. Results

Study recruitment took place from 27 June 2021 to 30 September 2021. The project started during the peak of the third COVID-19 wave in South Africa, which began in May 2021, where the average number of nationwide daily cases reached around 18,000, with a weekly test positivity rate of between 25% and 30% [33]. By the end of the project in September, the daily positivity rate for the country had fallen to below 5%, coinciding with the end of the third wave [33]. 

Overall, 15,443 participants were screened into the study. Of the total participants, 81.8% (n = 12,638) were commuters at the taxi ranks, 4.0% (n = 611) were drivers and 2.4% (n = 373) were vendors (Table 1). Another 11.8% of participants were listed as “other”—this included individuals who had come to the taxi rank to shop, or specifically to access the testing service. The median age of all participants was 32 years, and around half of all participants (48.9%) were female.

### 3.1. Primary Objective: Risk Assessment, Testing and Reporting

**Figure 2:**
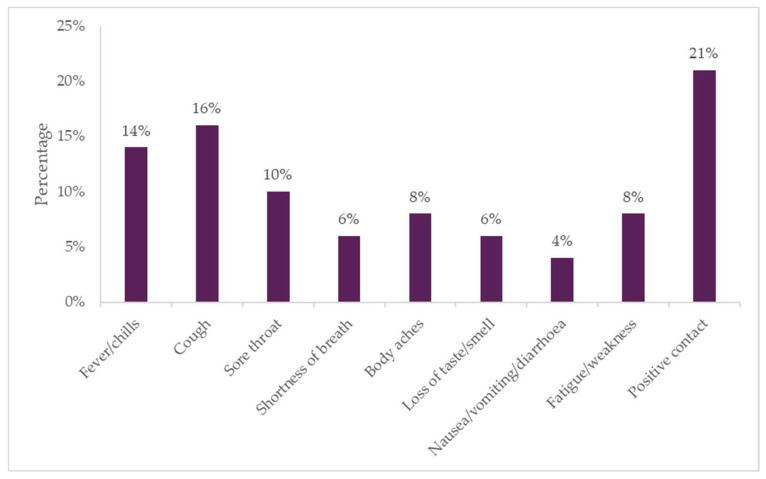
Percentage of participants reporting COVID-19 risk factors. Positive contact refers to close contact with a known COVID-19 positive individual.

Of the 15,443 participants screened as part of the study, most were classified having a low risk of COVID-19. Overall, 19% (n = 2987) participants who underwent screening were classified as “high risk” (2 or more risk factors); 13% (n = 2061) screened were classified as “moderate risk” (at least 1 risk factor), while 67% (n = 10,395) were “low risk” (no risk factors). Figure 2 shows the percentage of participants reporting COVID-19 risk factors (as per screening checklist). 

**Figure 3:**
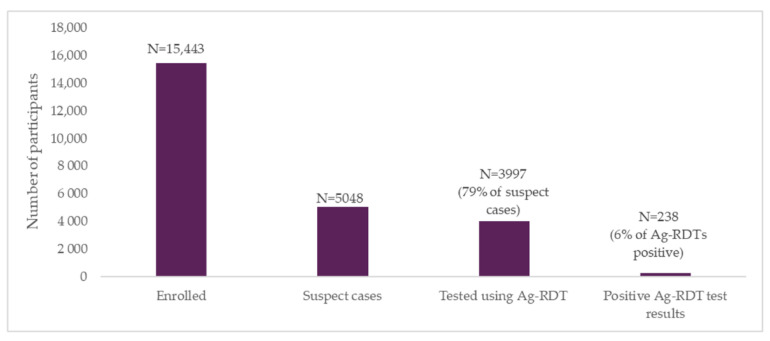
Suspect cases and SARS-CoV-2 testing at enrollment.

Overall, 5048 individuals (n = 2987 high risk + 2061 moderate risk) were classified as having a high to moderate risk of COVID-19. This group, comprising 33% of the overall screened population were eligible for testing with the Ag-RDTs. Of the individuals eligible for Ag-RDT testing, 79% (n = 3997) received a test—85% of high-risk participants, and 69% of moderate-risk participants (Figure 3). A further 1% of low-risk individuals also elected to receive an Ag-RDT.

Results were sent to participants by both SMS and WhatsApp message. Of the 3997 participants who completed Ag-RDTs and were sent results by WhatsApp, 63% of messages were delivered and 90% of these messages were marked as read. The actual read rate may be higher as WhatsApp has a feature that enables messages to be read without notifying the recipient. It was not possible to monitor read rate for participants who received their results by SMS only. 

### 3.2. Secondary Objectives

#### 3.2.1. Digital Follow up of Low-Risk Participants and Those Testing Positive on Ag-RDT

Of the 10,395 participants that were identified as low risk, 690 individuals (6.7%) completed at least one phone-based follow-up survey. Of the 239 participants with a positive Ag-RDT result, 200 (84%) completed the 10-day post-diagnosis follow up questionnaire indicating a high willingness to follow up via the use of digital tools.

Of the 5048 suspected cases eligible to get tested, 1013 participants who were eligible for testing did not get tested. A higher proportion of participants who screened “high-risk” (those with a greater number of risk factors for COVID-19) went on to get tested (85%) than those who screened “moderate-risk” (69%). The main reasons given for not following through with testing were time (8% reporting they had to wait too long, or had somewhere else to be). Wait times initially averaged 45 min but were reduced to 20 min through adding study team resources and increasing the size of the team from one nurse to two nurses. 

Several participants were also unwilling to complete the study once learning no compensation would be provided—in contrast to several other COVID-19 studies in nearby locations that did offer compensation in order to incentivize participation. In addition, adverse weather conditions (i.e., rain, high winds) affected workflow thereby also deterring participation.

#### 3.2.2. SARS-CoV-2 Positivity Rate for Suspect Cases Tested Using Ag-RDTs

Out of the 3997 Ag-RDT tests performed, 6.0% of tests were positive for SARS-CoV-2. Positivity rate was highest among participants in the high-risk group (5.1%), and considerably lower among participants in the moderate (0.8%) and low-risk groups (0.1%). Across occupations, commuters had the highest proportion of positive Ag-RDTs results (4.3%), followed by participants with “other” occupations (1.5%), while positivity rates were much lower among drivers (0.1%) and vendors (0.1%). Among moderate-risk participants that were asymptomatic and reported coming into contact with a positive COVID-19 case, positivity rate was also low (1%). 

#### 3.2.3. SARS-CoV-2 Positivity Rate among Randomly Selected Individuals Tested Using PCR

Of the 3997 individuals that received an Ag-RDT, 582 (14.6%) also received a PCR test. Of the 582 PCR tests performed, 17.5% were positive for SARS-CoV-2. The positivity rate was 14.9% among participants in the high-risk group, 2.4% among the moderate-risk group, and 0.2% among the low-risk group.

**Figure 4:**
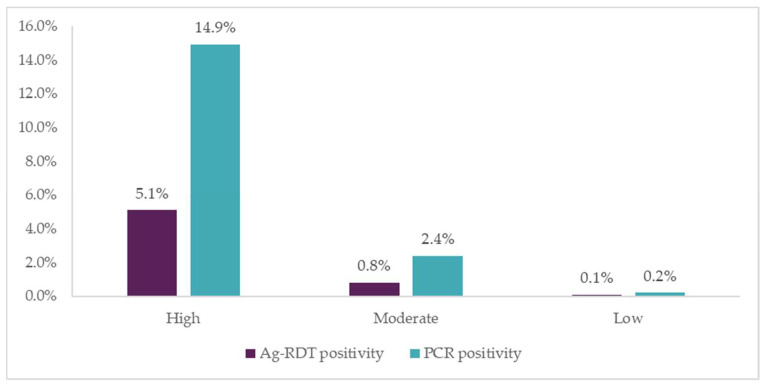
SARS-CoV-2 positivity rate for Ag-RDT and PCR tests by risk group.

As with the Ag-RDT results, commuters had the highest positivity rate on PCR tests (14.1%) followed by those with “other” occupations (3.1%), with similarly low positivity rates among drivers and vendors (0.2% each). Figure 4 shows these SARS-CoV-2 positivity rates, for both Ag-RDT and PCR tests, broken down by risk group.

Although overall positivity rate was higher with the PCR tests, the similar pattern in positivity rates by risk category for Ag-RDT and PCR testing indicates that the digital tool performs well in terms of predicting those at highest risk of SARS-CoV-2 infection (Figure 4).

#### 3.2.4. Field Performance of Ag-RDTs in the Study

Out of the 569 individuals who received both Ag-RDT and PCR testing, seven individuals who tested positive with the Ag-RDT were negative by PCR test (false positive results). A further 60 individuals had false negative results (negative Ag-RDT, but positive PCR). Tests were indeterminate for three individuals due to insufficient samples. 

Corresponding sensitivity and specificity values for the Ag-RDT were 40.0% (95% CI: 30.3%, 50.3%) and 98.5% (95% CI: 96.9%, 99.4%), respectively. The positive predictive value of the Ag-RDT in this setting was 85.1% (95% CI: 71.7%, 93.8%), and the negative predictive value was 88.5% (95% CI: 85.5%, 91.1%). 

A breakdown of positive PCR results by cycle threshold (Ct), which is a proxy for SARS-CoV-2 concentration or viral load, was retrieved for each positive PCR result and reported for each gene target (*N* gene, *orf1ab* and *S* gene). This analysis found that the median Ct value for true positives detected by Ag-RDTs was <20 (high viral load), while the majority of false negatives had Ct values >25 (low viral load) (Supplementary Figure S2).

Using the end-to-end testing data captured in the digital platform, the median *N*-gene Ct values were further broken down by symptom duration. This analysis found that the highest proportion of false negatives was among participants who reported that they had experienced symptoms for 3–5 days (21.4%), most of whom had Ct values over 25. 

When only samples with a Ct cutoff of 30 were included in the analysis, which is the range under which Ag-RDTs are evaluated in South Africa, sensitivity of the Ag-RDT increased to 59.1% (95% CI: 49.3%, 71.1%) (Table 2).

#### 3.2.5. Participants’ Level of Satisfaction with the Digital Solution in this Context

Of the participants who were tested, a randomly selected group of 161 participants received a digital questionnaire after the intervention, enquiring about their level of satisfaction with the digital solution. Response late was low, with only 25 (6%) submitting feedback. Participants could rate their satisfaction on a scale of 1 to 4, with 1 being “Very Happy” and 4 being “Not happy”. Of the group, 72% and 12% reported that they were “very happy” and “happy”, respectively. However, this sample was too small to draw conclusions.

#### 3.2.6. Commuter Perspectives and Practices on SARS-CoV-2 Testing

##### Baseline Survey

Among 1515 respondents randomly drawn from commuters in the taxi rank prior to the testing intervention, most individuals (79%) had not had a previous COVID-19 test. Among the 21% of individuals who had been tested previously, 67% did not pay for the test. Although most individuals (85%) preferred to get tested at a private doctor/clinic, 84% of respondents said they would be willing to get a COVID-19 test at or near a taxi rank if made available.

##### End-Line Survey

Among the 1144 respondents randomly drawn from commuters in the taxi rank after the intervention was completed, the proportion of commuters who had previously been tested for COVID-19 increased from the baseline to end-line survey, with 67% of participants in the end-line survey having been tested at least once. 

Of survey respondents that had at least one test in the past, 51% (n = 389) had an Ag-RDT, 13% (n = 96) had a PCR test, while 37% (n = 281) had both types of test. Among respondents who had been previously tested, the majority (67%, n = 519) had been tested as part of a research study. Nearly all (99%) of those who had received at least one COVID-19 test in the past had some level of schooling. 

Of those who had at least one test, 40% said they took the test because they had symptoms of COVID-19, 16% came into contact with someone who had COVID-19, 15% needed a negative COVID-19 test for work, school, or travel, and 15% just wanted to know their status. 

Of the 1114 participants who reported a willingness to get tested, 85% (n = 951) would be willing to get tested in a decentralized location, including at school, work or near a taxi rank, as long as the location provided privacy. Although willing to get tested, 70% of this group would not be willing to pay for a test.

## 4. Discussion

### 4.1. Expanding Access to Point-of-Care Testing for COVID-19

Access to COVID-19 testing when and where people need it is vital to controlling the pandemic. Travel to public sector health facilities can pose a barrier to testing, due to associated costs and lack of patient-friendly services [34]. Bringing screening and diagnosis closer to the patient is key to expanding access [35] and can help limit transmission of infectious diseases by limiting congestion in facilities. 

This study demonstrated that community-based decentralized COVID-19 testing in heavy foot traffic areas can detect large numbers of at-risk people in a short amount of time. Despite various barriers to recruitment throughout the study, discussed later, enrolment numbers exceeded targets, with 115% of the projected sample size of participants registering for screening. A baseline survey of randomly selected commuters revealed that the majority of respondents (79%) had not been previously tested for COVID-19, but would be willing to get tested if services were available at or near high foot traffic locations such as transport hubs i.e., taxi ranks. The end-line survey showed an increase in the proportion tested from 21% to 67%, with 51% of respondents who had been previously tested having received an Ag-RDT, indicating the critical role of point-of-care diagnostics in expanding access to testing. Similarly, 85% of end-line survey respondents said they would be willing to get tested in a decentralized setting.

### 4.2. Digital Tools to Facilitate Decentralized Access to Testing

Data management is a common concern associated with scaling up point-of-care COVID-19 testing. The accurate capturing and rapid transmission of data is central to an effective pandemic response, and can be difficult to ensure when expanding testing, especially outside of established health facilities. This study demonstrated that digital technologies can effectively support case management, as well as real-time data capture, transmission and analysis for testing services deployed in community settings. Fieldworkers reported that the digital tool made screening easy and less time-consuming compared with paper-based forms. The use of delta-checks, a quality checking system, during the data capture process provided critical quality assurance measures to ensure the high quality of data. In addition, the digital tool did not allow for incomplete fields and used machine-guided logic for drop-down menus and correct spelling, which aided in accurate and standardized reporting.

Importantly, the digital tool also enabled rapid transmission of results to participants, with delivery receipts available for at least two thirds of participants and 90% of delivered messages being fully delivered. It also facilitated follow-up of two key participant groups of interest: those who tested positive for SARS-CoV-2, and those who dropped off between screening and testing. Around 84% of individuals with positive Ag-RDT results completed the 10-day post-diagnosis follow-up questionnaire for self-monitoring of symptoms and clinical outcomes, with no severe events reported. This indicates strong adherence and willingness to use digital tools. The comprehensive data captured across the testing continuum provided end-to-end visibility on the patient pathway, and allowed for more granular analysis of both operational and epidemiological metrics documented in the study.

A limitation of the study was the inability to confirm receipt of test result messages for participants who did not have WhatsApp. If using digital devices to deliver results, it may therefore be necessary to develop alternative means to confirm that results have been received. Examples of approaches from previous studies using mobile-based results delivery include phone calls to SMS recipients to confirm receipt of the message [36], but given how resource-intensive this approach is, it is not feasible to implement on a large scale. An alternative approach could be to request that participants send a one-word text to confirm results receipt, and if this is not received, to send a limited number of reminders within a pre-defined timeline.

### 4.3. Screening and Testing Outcomes

Out of around 15,000 participants who enrolled in the study, 2061 (13%) were classified as moderate risk, and 2987 (19%) as high risk, based on the digital screening algorithm. Of these participants who were eligible for testing, around 1 in 5 subsequently dropped off before being tested, and were followed up telephonically. A total of 3997 moderate- and high-risk participants went on to receive testing with Ag-RDTs and had their results sent automatically to their phones through the digital tool. 

Positivity rates were highest among people in the “high-risk” group (two or more COVID-19 risk factors), followed by those in the “moderate-risk” group (one COVID-19 risk factor). This suggests that when deploying testing in high-volume sites, a simple risk-scoring algorithm based on symptoms and contact history can effectively identify people to prioritize for testing, allowing for more efficient use of resources. However, if new SARS-CoV-2 variants present different symptomatic patterns, the usefulness of such an algorithm may change.

Another limitation of this algorithm is that it may miss asymptomatic patients who are not aware of their recent exposure to a confirmed COVID-19 case. The study therefore permitted individuals who were categorized as low risk, but still wanted testing, to receive an Ag-RDT. Ag-RDT positivity rate was lowest in this group (0.1%), but a handful of cases were still detected. Among moderate risk cases who were asymptomatic but had contact with a COVID-19-positive patient, 1% were positive by Ag-RDT, highlighting the prevalence of asymptomatic COVID-19 in the community. 

These findings are also insightful as symptom data are not routinely reported as part of COVID-19 testing outcomes. Systemic use of end-to-end digital tools as employed in this study can help to better understand the epidemiology of asymptomatic infection by connecting testing outcomes to symptoms captured earlier in the continuum. For example, the Surveillance Outbreak Response Management and Analysis System (SORMAS), used by the Nigeria Centre for Disease Control for COVID-19 management, indicated early in the pandemic that over half of cases confirmed using PCR were asymptomatic at the point of diagnosis [37]. However, more evidence is needed to inform resource-efficient approaches around the inclusion of asymptomatic individuals in COVID-19 testing strategies.

### 4.4. Field Performance of Ag-RDTs

There was also concordance between PCR and Ag-RDT positivity rates, particularly among patients with higher viral loads as indicated by lower Ct values. Positivity was higher with the PCR tests, as expected for the “gold standard” test with higher accuracy. Our findings also suggest that Ag-RDTs can serve as a good indicator of infectiousness, as the highest positivity rate was found in high-risk symptomatic participants. However, further multivariate analysis is needed to understand the relationship between symptom type and duration with Ag-RDT performance. 

The sensitivity of the Ag-RDT in the study was 40.0%, lower than estimates from meta-analyses of Ag-RDT sensitivity, where pooled sensitivity of Ag-RDTs in symptomatic individuals was 72.0% and 76.7%, respectively [11,12]. However, these analyses have also identified a considerable loss of Ag-RDT sensitivity in individuals with higher Ct values (50.7% for Ct ≥ 25 [12] and 40.7% for Ct > 25 [11]). Nevertheless, the positive and negative predictive values of the Ag-RDT in this setting, which are dependent on the prevalence of COVID-19 in the population, were higher at 85.1% and 88.5%, respectively. Consequently, when used in a setting with a relatively high prevalence of COVID-19, a lower sensitivity Ag-RDT may still be of value from a public health perspective, particularly if combined with targeted confirmatory PCR testing [8].

For example, in settings with a high prevalence of COVID-19, it may be advisable for all individuals with a high risk of COVID-19 to receive confirmatory PCR testing if their Ag-RDT is negative. This approach is recommended in many LMICs, including South Africa. South Africa’s national Ag-RDT guidance at the time the study was implemented recommended confirmatory PCR testing for those with a “high pre-test probability of a positive result” who test negative by Ag-RDTs. 

However, in the absence of more specific guidance, it is difficult to implement this consistently. Overly broad interpretation of what constitutes a “high risk” patient could result in increased demand for PCR testing that undermines the value of decongesting central laboratory systems through the use of Ag-RDTs. On the other hand, given high rates of community transmission, especially with the Omicron variant and under-detection of COVID-19, individuals with recent exposure to a case may not be aware of it and may thus not benefit from confirmatory PCR testing if they test negative with Ag-RDTs. 

These challenges highlight the value of routine end-to-end data capture when delivering testing services, including capturing patients’ symptoms and other clinical factors, to identify the most important predictors of infection that can be recognized at the point of care. The application of digital tools to capture such data, and potentially of algorithms to identify individuals for whom confirmatory testing would be most beneficial, could help to optimize use of resources and inform a standardized approach. For example, in this study, low blood oxygen levels showed the strongest correlation with infection, although further multivariate analyses would be needed to confirm this correlation. However, given the unpredictable characteristics of SARS-CoV-2 infection as new variants emerge, guidance on selection for confirmatory testing may need to be determined on a case-by-case basis and frequently updated. 

### 4.5. Importance of Transport Hubs for COVID-19 Testing

In this study, transport hubs were selected for the testing intervention with Ag-RDTs and the digital tool for being both high-traffic and high-transmission settings. The study identified a high percentage (22%) of people at the taxi ranks who had at least one symptom related to COVID-19. As the majority of participants were commuters, drivers and vendors present at the taxi rank as part of their normal routine, the findings shed light on how many potentially COVID-19-positive individuals are present in everyday community settings, and the value of increasing access to decentralized COVID-19 testing. 

Importantly, the project managed to reach taxi drivers, providing over 600 drivers with COVID-19 screening as part of the study. Due to the busy schedules of taxi drivers, this group is often neglected, and considered hard to reach [38]. Considering that a single driver can transport over 200 people in a day, it is critical that drivers have access to testing services to detect COVID-19 early and take measures to prevent potential transmission to passengers. 

The overall positivity rate reported throughout the three months was around 6.0% using Ag-RDTs, peaking at 22% during the third wave. Among the subgroup of participants who received PCR testing, average positivity rate was three times higher (17.5%), which is similar to the average positivity rate reported in Gauteng province during the same period (17%) [33]. Assuming community-based testing models correlate with positivity rates in the broader geographic setting, they could also be considered as a monitoring strategy for early detection of epidemic waves.

### 4.6. Participant Follow-Up and Observations 

While a high response rate was noted for digital follow-up with participants who tested positive, this was not the case for those who screened low risk, with only 7% completing a follow-up survey, indicating a low perception of risk was linked to lower engagement with follow-up. Among those who were eligible for testing but didn’t receive an Ag-RDT, study personnel were able to contact 64% (n = 701) out of 1103 participants who dropped off. Long waiting times were the most common reason provided by participants for not completing the study. Subsequently, in future testing projects, efforts to increase workflow efficiencies and decrease waiting times should be a priority. As adverse weather conditions also deterred participation, the use of more solid structures able to withstand different weather conditions would also be useful in future outdoor testing projects. Furthermore, given the high percentage of survey participants who said they would be unwilling to pay for a COVID-19 test, this study highlights the importance of making sure testing is available for free—and clearly advertised as freely available to encourage uptake.

### 4.7. Operational Findings

The real-time data capturing and dashboard performance assessments on the digital tool enhanced visibility into operational details and allowed for quick identification of and responses to any issues. We found that a team comprising two nurses, two research assistants and three fieldworkers worked well for each site, and enabled screening of around 40 people per hour. As expected, testing sites were busiest during peak hours at the taxi rank (early morning, lunch, and late evening). 

Challenges encountered during the study included that some participants, particularly taxi drivers, were hesitant to know their COVID-19 status due to fear of discrimination at work or loss of income if found positive. Some participants were also nervous about nasopharyngeal swabbing for the tests. In future testing projects, additional community engagement and awareness activities would be helpful to reduce stigma around testing and highlight the benefits of getting tested. Some participants also reported that the testing tents did not offer sufficient privacy, so utilizing mobile testing booths that allow for greater privacy may be a better option in the future, provided appropriate infection control measures are used (e.g., ensuring adequate ventilation). 

## 5. Conclusions

Community-based decentralized COVID-19 testing initiatives can improve access to testing for individuals, households and workplaces, making it easier for people to know their COVID-19 status and subsequently make informed choices about exposure risk. This study demonstrates the value of offering COVID-19 testing in strategic community locations such as busy taxi ranks, to reach members of the community who may not have easy access to testing. The use of the digital tool combined with Ag-RDTs in this setting was found to enhance service delivery by providing standardized screening, enabling real-time data capture and transmission, and facilitating participant follow-up. Overall, our findings indicate that digital health solutions combined with rapid point-of-care testing can be used to improve early COVID-19 surveillance and response efforts, and importantly improve patient-centred care, by making testing more accessible.

## Figures and Tables

**Table 1:** Demographic characteristics of participants by study site.

Study Site				
Variable	Baragwanath	Germiston	Randburg	Total
Enrolled, n (%)	5548 (35.9%)	5849 (37.9%)	4046 (26.2%)	15,443 (100.0%)
Occupation, n (%)				
Commuter	4754 (85.7%)	4447 (76.0%)	3437 (84.9%)	12,638 (81.8%)
Driver	244 (4.4%)	300 (5.1%)	67 (1.7%)	611 (4.0%)
Vendor	116 (2.1%)	190 (3.2%)	67 (1.7%)	373 (2.4%)
Other	434 (7.8%)	912 (15.6%)	475 (11.7%)	1821 (11.8%)
Sex, n (%)				
Female	2852 (51.4%)	2584 (44.2%)	2109 (52.1%)	7545 (48.9%)
Not female	2696 (48.6%)	3265 (55.8%)	1937 (47.9%)	7898 (51.1%)
Age (years)				
Median (IQR)	33.0 (10)	34.0 (12)	30.0 (10)	32.0 (11)

**Table 2:** Performance of the Ag-RDT compared with a PCR reference standard, with a Ct cut-off of 30.

Ag-RDT Performance (at Ct Cut-Off = 30)	
Sensitivity (95% Cl)	59.1% (49.3, 71.1)
Specificity (95% Cl)	98.7% (97.2, 99.5)
Positive predictive value (95% Cl)	85.1% (71.7, 93.8)
Negative predictive value (95% Cl)	88.8% (85.7, 91.4)
Cohen’s Kappa (95% Cl)	0.50% (0.39, 0.60)

## Data Availability

The data that support the findings of this study are available on request from the corresponding author.

## Supplementary Materials

The following supporting information can be downloaded at: https://www.mdpi.com/article/10.3390/diagnostics12020402/s1, Figure S1: Screening checklist; Figure S2: Cycle threshold breakdown by gene target for positive laboratory PCR results.
