# Supplementary material for: Monitored Implementation of COVID-19 Rapid Antigen Screening at Taxi Ranks in Johannesburg, South Africa
Source: Diagnostics (Basel). 2022 Feb 3;12(2):402. doi: 10.3390/diagnostics12020402 (PMC8871379; doi:10.3390/diagnostics12020402)
Supplement: Supplementary file 1 [file diagnostics-12-00402-s001.zip › diagnostics-1558398-supplementary.pdf]

Date: DD/MM			
Symptoms (answer Y or N)	**Weekly/more often if desired**		
Fever/Chills	Y	/	N
Cough	Y	/	N
Sore throat	Y	/	N
Shortness of breath	Y	/	N
Body aches	Y	/	N
Loss of smell OR loss of taste	Y	/	N
Nausea/vomiting/diarrhoea	Y	/	N
Fatigue/weakness	Y	/	N
Close contact with known Positive	Y	/	N

Figure S1. Screening checklist.

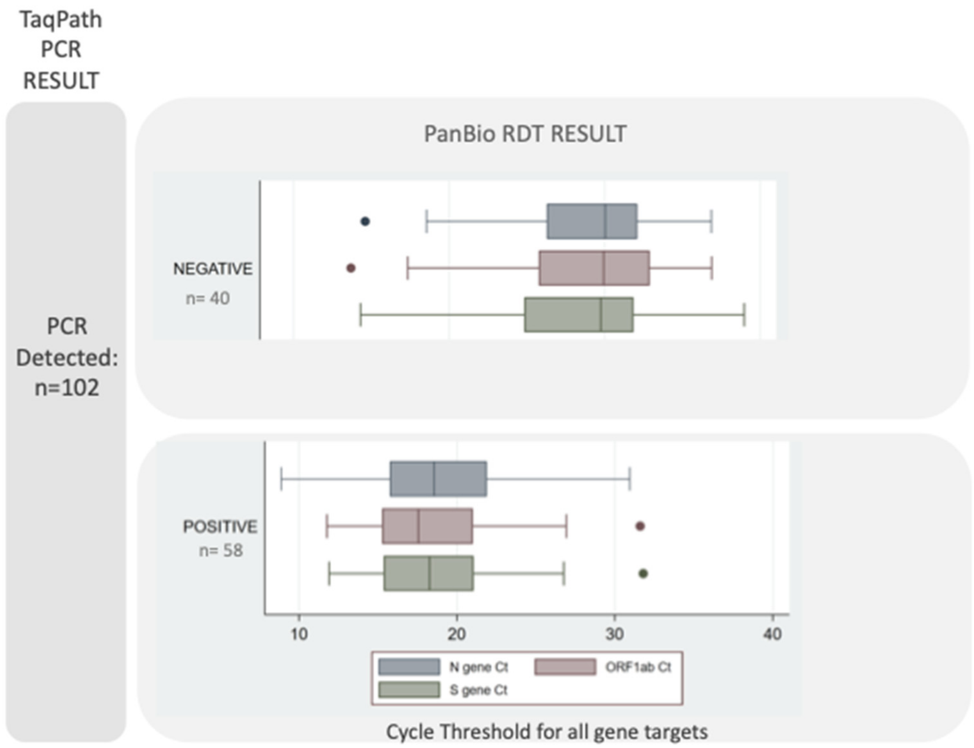

Figure S2. Cycle threshold breakdown by gene target for positive laboratory PCR results.
